# Mr.Vc v2: An updated version of database with increased data of transcriptome and experimental validated interactions

**DOI:** 10.3389/fmicb.2022.1047259

**Published:** 2022-11-22

**Authors:** Zhiyuan Zhang, Guozhong Chen, Wajid Hussain, Zixin Qin, Juntong Liu, Yang Su, Hao Zhang, Mingquan Ye

**Affiliations:** ^1^School of Medical Information, Wannan Medical College, Wuhu, China; ^2^Research Center of Health Big Data Mining and Applications, Wannan Medical College, Wuhu, China; ^3^College of Life Sciences and Technology, Huazhong University of Science and Technology, Wuhan, China; ^4^Advanced Biomaterials and Tissue Engineering Center, College of Life Sciences and Technology, Huazhong University of Science and Technology, Wuhan, China

**Keywords:** update, interactions, transcriptome, text-mining, Top 5% DEG, *Vibrio cholerae*

## Abstract

Mr.Vc is a database of curated *Vibrio cholerae* transcriptome data and annotated information. The main objective is to facilitate the accessibility and reusability of the rapidly growing *Vibrio cholerae* omics data and relevant annotation. To achieve these goals, we performed manual curation on the transcriptome data and organized the datasets in an experiment-centric manner. We collected unknown operons annotated through text-mining analysis that would provide more clues about how *Vibrio cholerae* modulates gene regulation. Meanwhile, to understand the relationship between genes or experiments, we performed gene co-expression analysis and experiment-experiment correlation analysis. In additional, functional module named “Interactions” which dedicates to collecting experimentally validated interactions about *Vibrio cholerae* from public databases, MEDLINE documents and literature in life science journals. To date, Mr.Vc v2, which is significantly increased from the previous version, contains 107 microarray experiments, 106 RNA-seq experiments, and 3 Tn-seq projects, covering 56,839 entries of DEGs (Differentially Expressed Genes) from transcriptomes and 7,463 related genes from Tn-seq, respectively. and a total of 270,129 gene co-expression entries and 11,990 entries of experiment-experiment correlation was obtained, in total 1,316 entries of interactions were collected, including 496 protein-chemical signaling molecule interactions, 472 protein–protein interactions, 306 TF (Transcription Factor)-gene interactions and 42 *Vibrio cholerae*-virus interactions, most of which obtained from 402 literature through text-mining analysis. To make the information easier to access, Mr.Vc v2 is equipped with a search widget, enabling users to query what they are interested in. Mr.Vc v2 is freely available at http://mrvcv2.biownmc.info.

## Introduction

Cholera is a notorious and devastating diarrheal disease, until now had caused seven epidemics in history globally and is still endemic in many parts of the world, especially developing countries like Asia, South America, and Africa (Faruque et al., 1998; Liu et al., 2008; Xia et al., 2017).

*Vibrio cholerae* is the causative agent of cholera resulting in 23,000 to 143,000 people dying worldwide annually (Qin et al., 2020). However, the pathogenesis of *Vibrio cholerae* is still unclear. Growing evidence suggests that *Vibrio cholerae* can rapidly modulate its gene transcriptional expression in response to the switches of different environments for better survival and infection. Therefore, studying the gene expression profile of *Vibrio cholerae* under various conditions is important to fully expose and understand the genetic mechanism of *Vibrio cholerae.*

In recent years, the transcriptional sequencing is powerful enough to study general gene expression profiles, sequencing of *Vibrio cholerae* transcriptomes rapidly increased the number and total volume of *Vibrio cholerae* transcriptome data. At present, most of the raw sequencing data has been deposited into several general-purpose databases, such as European Nucleotide Archive (ENA) (Amid et al., 2020)^1^ and NCBI Sequence Read Archive (SRA) (Kodama et al., 2012).^2^ In addition, several other public resources, including MicrobesOnline (Dehal et al., 2010), PubMLST (Jolley et al., 2018), BioCyc (Paley and Karp, 2017), and PATRIC (Wattam et al., 2014), collected the processed transcriptome data, microbial genome and metabolic pathway information and then organized them according to experimental conditions and organisms for the purposes to greatly promote data reuse. However, obstacles to the reusability and accessibility of the rapidly growing *Vibrio cholerae* transcriptome data remain, especially the inaccurate data sets and/or incomplete data. For example, MicrobesOnline, which integrated vast amounts of microbial genetic information, did not update transcriptome data since 2012 and only collected 42 high-throughput *Vibrio cholerae* microarray data, not collecting RNA-seq data, under different experimental conditions, deriving from seven published papers. Additionally, MicrobesOnline did not compute *p*-values in differential gene expression analysis due to lacking technical/biological replicates. PubMLST, which integrated population sequence data with provenance and phenotype information for over 100 different microbial species and genera, focus on the analysis of molecular typing and microbial genome diversity, and did not collect microbial transcriptome data, including *Vibrio cholerae.* BioCyc, a database for collection of the genome and metabolic pathways of organisms, provided annotations, essentiality and reactions of gene or protein, however, transcriptome data were not considered for collection in BioCyc.

Although identified DEGs from transcriptome data are useful to discover novel genes for phenotypes, obtaining a comprehensive regulatory network is meaningful (Saint-André, 2021). To date, several databases, including STRING (Szklarczyk et al., 2021), CollecTF (Kiliç et al., 2014), STITCH (Szklarczyk et al., 2016), and MVP (Gao et al., 2018), had provided data of protein–protein, TF (Tanscription Factor)-gene, protein-chemical signaling molecule, and *Vibrio cholerae*–virus interactions. These data will better help to understand *Vibrio cholerae* and useful for researchers to work on *Vibrio cholerae*. However, some interaction data presented in databases were obtained through prediction rather than experimental verification, which cannot ensure the data quality.

In 2019, we introduced Mr.Vc v1 as an online database of curated microarray and RNA-seq of *Vibrio cholerae* to facilitate the reusability and accessibility of the rapidly increasing *Vibrio cholerae* omics data and relevant annotation (Zhang et al., 2019). We collected data from 145 high-throughput gene expression experiments of *Vibrio cholerae* from 49 journal articles and the detailed annotation for 3,834 genes of *Vibrio cholerae* (*Vibrio cholerae O1 biovar eltor str. N16961*), we also collected relevant information including which operons they may belong to and possible interaction partners of their protein products. Mr.Vc is the first comprehensive data repository dedicated to *Vibrio cholerae* and could provide convenience for all researchers in related fields.

In current study, we are going to introduce an updated version of Mr.Vc. In this new version, we extended more annotation information, such as operon annotation through text-mining analysis, collected more transcriptome projects, samples, experiments, and performed extensive analysis, including differential gene expression analysis, operon member expression visualization, gene co-expression analysis, and experiment-experiment correlation analysis. Most importantly, we added an “Interactions” functional module in Mr.Vc v2, which listed collected experimentally validated interaction entries about *Vibrio cholerae*. We obtained them from public databases including STRING, STITCH, CollecTF, MVP, literature-describing interactions referring to *Vibrio cholerae*, and text-mining results from MEDLINE documents and literature in life science journals. Additionally, we manually categorized these interaction data into four interaction types, including protein-chemical signaling molecule interaction, protein–protein interaction, TF (Transcription Factor)-gene interaction, and *Vibrio cholerae*-virus interaction. These interaction data will provide us with more clues into the regulatory network and mechanism in *Vibrio cholerae* (Kathuria and Chattopadhyay, 2018). Mr.Vc v2 is equipped with a search widget, allowing experimental biologists and medical scientists to quick and easy finding what they are interested.

## Materials and methods

### Data collection and pre-processing

**Figure 1:**
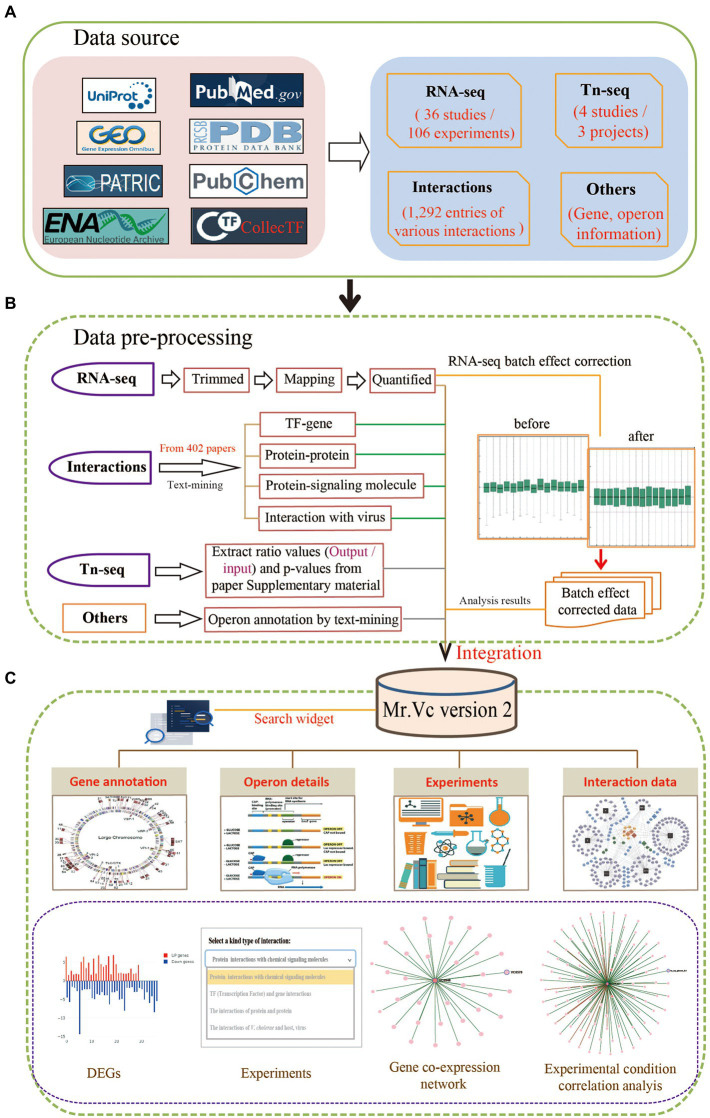
Overview of Mr.Vc v2. **(A)** The data source. All data including the raw sequencing data, information on genomic annotation, literature, Tn-seq data, and experimentally validated interactions were collected from public databases, such as NCBI GEO, ENA, PATRIC, Uniprot, collecTF, PDB, PubMed, and so on. **(B)** The data pre-processing. The RNA-seq raw data perform quality control, mapping, transcript quantification, and normalization to obtain high-quality data for further analysis. The resulting operon and interaction data were obtained from MEDLINE documents and literature in life science journals through text-mining analysis. The Tn-seq data were directly extracted from supplemental materials of literature. **(C)** The data integration. Mr.Vc v2 integrated polytype data, which was placed in the “Genes,” “Operons,” “Experiments,” and “Interactions” pages and provided a search widget.

To give users a clear overview of the data collection, pre-processing, and integration in Mr.Vc v2, we provided the detailed workflow (Figure 1).

For data collection, as shown in Figure 1A, we searched recently updated RNA-seq projects in the NCBI BioProject database^3^ and publications in NCBI PubMed^4^ using “*Vibrio cholerae”* as the keyword. Projects with public raw sequencing data, 106 RNA-seq datasets were collected for further analysis. The raw sequencing reads were downloaded from EBI ENA (European Nucleotide Archive,^5^) and NCBI SRA (Sequence Read Archive,^6^) using command line tools from enaBrowserTools^7^ and SRA-Tools^8^ facilitated by Aspera (a high-speed data transfer tool). As the related meta-data of corresponding experiments, projects and literature were obtained from NCBI PubMed and GEO databases.

For the processing of raw sequencing reads, we used FastQC^9^ to evaluate the overall quality of the downloaded data, followed by the Trim_galore to remove sequencing vectors and low-quality bases (Utturkar et al., 2020). Salmon is the latest computational algorithm for transcript quantification from RNA-seq data that could make the expression data compared across experiments and projects (Patro et al., 2017). In this study, we performed transcript quantification using Salmon, which adopted TPM (Transcript Per Million) for normalization, a better unit for RNA abundance than RPKM and FPKM since it respects the invariance property and is proportional to the average relative RNA molar concentration (Zhao et al., 2020). We searched Tn-seq projects in the NCBI PubMed database and downloaded corresponding supplemental materials of literature. These data included experiment details and the ratio value of output/input.

We performed text-mining analysis for *Vibrio cholerae* operon annotation using a customized Python pipeline by searching for functional descriptions of operon member genes (e.g., gene symbol, locus ID, common name, and aliases) in the titles and abstracts of MEDILNE documents and literature in life science journals available from the NCBI PubMed database. Additionally, for each operon, we visualized the expression profile of its member genes using TPM (Transcript Per Million) values in 110 different experiments. For example, operon “OP437” is involved in the function of bacterial motility, whose member genes expression trends were intuitively observed through visualization^10^ that could help us deduce a positive or negative regulatory relationship between genes.

In addition, we also had a special focus on experimentally validated interactions about *Vibrio cholerae* in this study. We retrieved related interaction entries by following steps: (1) searching for literature with the keyword: “*Vibrio cholerae”*; (2) Filtering sentence from literature, and one sentence containing two of these: *Vibrio cholerae* gene name, chemical molecule name, and virus name. (3) This sentence should also contain one of the following words/phrases: *interaction, altered, associated, caused, confer, contribute, association, downregulate, elevate, implicated, increase, induce, influence, interact, involved, lead to, link, mediate, modulate, overexpressed, reduce, regulate, related, relationship, treat, binding, environmental signals, target, pathogenic, pathogenesis, progression, and transcriptional regulation.* (4) Extracting these sentences from literature as supporting evidence and finally identified 1,316 entries of different interaction types from 402 literature shown in (Table 1), including (1) a total 472 protein–protein interactions, all of which were experimentally validated; (2) 496 protein-chemical signaling molecule interactions that included protein information extracted from Uniprot and details of chemical molecule extracted from PubChem (Kim et al., 2021) and PDB database (Burley et al., 2017); (3) 306 TF-gene interactions, most of which obtained from collecTF database; (4) 42 *Vibrio cholerae* -virus interactions, including 30 entries extracted from literature and 13 from MVP database.

**Table 1:** Data summary in Mr.Vc v2 database.

Mr.Vc	Version 1	Version 2
Genes	3,834	3,998
Well-annotated operons	415	600
Literature	49	402
Transcriptome experiment	145	213
DEGs	25,937	56,839
Tn-seq projects	0	3
Top 5% differentially expressed genes	0	161
Experimentally validated interactions	0	1,316
Gene co-expression entries	0	270,129
Entries of experiment-experiment correlation	0	11,990
Text-mining analysis	No	Yes
Data visualization	No	Yes

### Data analyses

We analyzed transcriptome data and out of a total 106 RNA-seq experiments, 318 samples, in which the expression abundances were normalized as TPM values. For RNA-seq experiments, we used a cutoff of |log2 FC| > 1.5 (FC, fold change) and *p*-value <0.05 to define differentially expressed genes between experiments. The 33,180 differentially expressed gene entries were extracted, representing *Vibrio cholerae* gene expression under 106 different experimental conditions. We performed gene co-expression analysis and experiment-experiment correlation analysis using an in-house Python script to calculate pearson’s correlation coefficients, spearman’s correlation coefficients, and *p*-value between genes and between experiments, separately. Pearson’s correlation coefficient have a greater statistical power than spearman’s coefficients, however which requires that the statistical data should conform to normal distribution. Transcriptome data tend to conform to negative binomial distribution, causing poor reliability of pearson’s correlation coefficient analysis. And if using spearman’s coefficients, the statistical power is not good. So we provided both spearman’s coefficients and pearson’s correlation coefficients for complementation on Mr.Vc v2 database. We used a cutoff of pearson or spearman >0.8 and *p*-value <0.05 to filter data of correlation, 270,129 gene–gene correlation entries and 11,990 experiment-experiment correlation entries were obtained. In order to make more comprehensive use of these two coefficients of correlation, we calculated their average value for users’ reference. In addition, we calculated the *p*-values using pearson’s and spearman’s coefficients, respectively, and then compared the calculated *p*-value, finally outputted the larger *p*-value (< 0.05) to page.

### Propose of “Top 5% differentially expressed gene” hypothesis

During the analysis of *Vibrio cholerae* transcriptome data, we found that some genes tended to be differentially expressed in most experiments. These observations indicated multiple functions of genes. To seek out these potential genes and their biological significance, we proposed a hypothesis that 5% of genes in bacteria are active in most conditions, which defined as “Top 5% differentially expressed gene.” We calculated the numbers of the experimental condition of each gene when differentially expressed in experiments, and ranked the corresponding gene by the calculated number of experimental conditions. Then, we calculated the number of “Top 5% differentially expressed gene” in *Vibrio cholerae* that used the product of 0.05 and numbers of gene except for essential genes, which is stable in gene expression and less biological significance, in total obtained 161 genes. Finally, according to the ranking result, we regarded the top 161 genes as “Top 5% differentially expressed gene.” For more details of “Top 5% differentially expressed gene,” please go to “Help” page.^11^

### Database design and implementation

Mr.Vc v2 was designed as a relational database. All data were loaded into a MySQL database. The frontend of the website was coded using JavaScript and HTML, while the backend was coded using PHP with a Slim framework to support queries to the MySQL database and provide representational state transfer (REST) application programming interfaces (APIs) for programmable access to our data. The AngularJS framework was used to bride the front- and back-ends. Echarts.js and plotly.js used for visualizations at the front end. The website hosted on an Apache server.

## Results and discussion

### Overview of Mr.Vc v2

In Mr.Vc v2, we updated gene and operon annotation information, including 3,842 protein-coding genes, 98 tRNAs, 16 rRNA, 42 pseudo genes, 161 Top 5% differentially expressed genes, and 600 well-annotated operons. We also provided external links to public databases such as KEGG (Kanehisa et al., 2017), NCBI Entrez Gene,^12^ OGEE (Cao et al., 2019), Uniprot (UniProt, Consortium, 2021) and MicrobesOnline to allow users to explore in more details of these genes and operons;

We analyzed 106 RNA-seq datasets and performed differentially expressed gene analysis, experiment-experiment correlation analysis, and gene co-expression analysis, in total obtaining 33,180 DEGs, 11,990 entries of experiment-experiment correlation, and 270,129 entries of gene co-expression. In addition, Mr.Vc v2 added 3 Tn-seq projects and 1,316 entries of experimentally validated interaction through public databases and text-mining analysis, including protein–protein interaction, protein-chemical signaling molecule interaction, TF-gene interaction, and *Vibrio cholerae*-virus interaction. All data shown in Table 1.

### Top 5% differentially expressed genes shared by experiments

**Figure 2:**
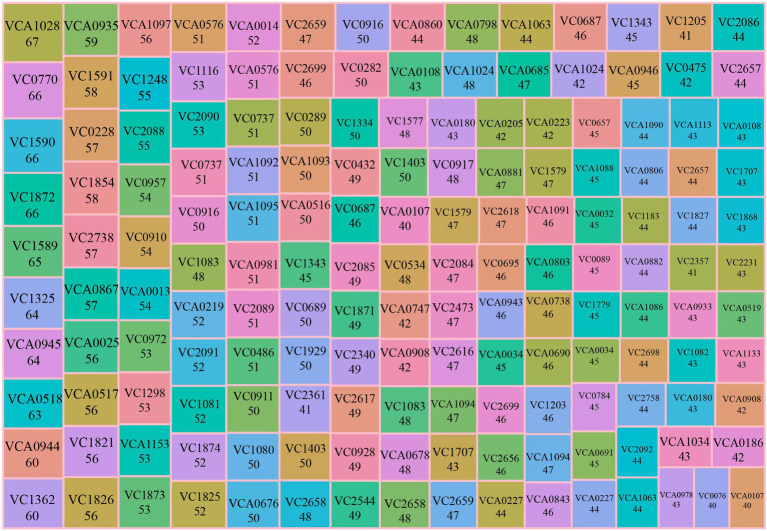
Summary of Top 5% differentially expressed genes. The treemap plot summarizes Top 5% differentially expressed genes. The number below the gene name is the number of experimental conditions that the gene was found to be differentially expressed, including up- and down-regulated. Mr.Vc v2 calculates the numbers of the experimental condition of each gene when differentially expressed in experiments, and ranks the corresponding gene by the calculated number of experimental conditions. A total of 161 Top 5% differentially expressed genes were obtained, according to ranking analysis. The Top 5% differentially expressed gene of the last rank was differentially expressed in 40 experimental conditions.

We found that experiments collected in our database shared some differentially expressed genes with other experiments, which suggested these genes might play an important role in multiple phenotypes. Therefore, the concept of “Top 5% differentially expressed gene” was proposed and according to the hypothesis, we ranked differentially expressed genes in 213 experiments based on the frequency of the gene’s appearance, in total obtaining 161 Top 5% differentially expressed genes (Figure 2). For example, Top 5% differentially expressed gene VCA1028, an outer membrane protein, was differentially expressed in 67 experiments (~31% of all), including gene deletion background, stress, nutritional condition, and human infection experiment. These data indicated this gene might be involved in several signaling pathways and functions. A previous study reported that VCA1028 was associated with virulence and indirectly regulated by ToxT (Thomson et al., 2015). However, there was no research to discuss its relationship with nutrition and stress. Therefore, the appearance of a “Top 5% differentially expressed gene” could remind researchers of its multi-function.

### Case study

**Figure 3:**
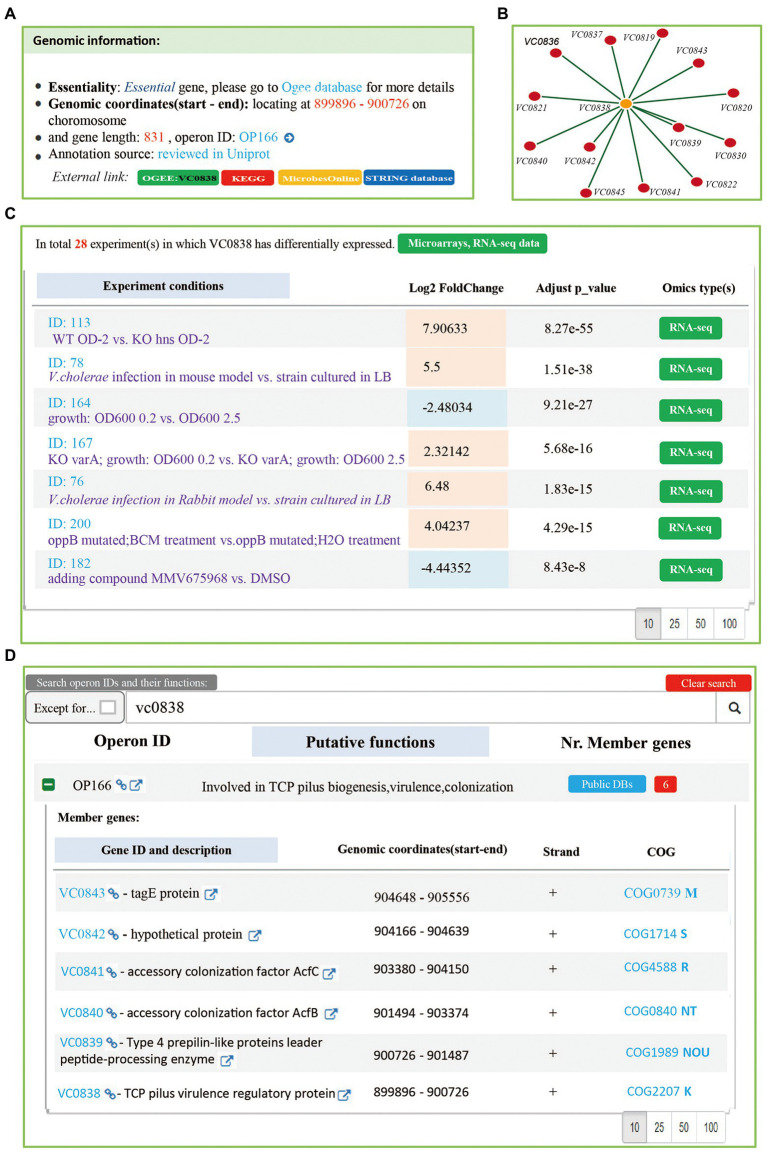
The “Genes and “Operons” page. **(A)** A summary of *Vibrio cholerae* gene VC0838. The information of gene annotation including gene ID, description, gene location, gene orientation, gene length, and gene essentiality was provided. **(B)** The gene co-expression network. Mr.Vc v2 filtered and obtained gene co-expression entries, according to the calculated pearson’s correlation coefficients (>0.8), Spearman’s correlation coefficients (>0.8), and *p*-value (<0.05) between genes. There are 14 genes associated with gene VC0838 in our database. **(C)** A list of experiments in which gene VC0838 is differentially expressed in. A total of 28 experiments were collected, including Microarray and RNA-seq experiments. **(D)** The operon information. Searching genes to match to corresponding operon page, which listed putative function and member genes information of operon.

In *Vibrio cholerae, toxT* and *tcpN* used to refer to the same gene (VC0838). In order to make users pain-free for these synonyms, we added “Aliases” data for genes.^13^ The *Vibrio cholerae toxT* gene (VC0838) was took as an example to represent how to use Mr.Vc v2 database for extraction of related information. The users have the access to five pages including “Genes,” “Operons,” “Experiments,” “Interactions” and “Download.” On the Mr.Vr v2 database functions, an additional page for instructions and a home page. On the “Genes” page, all gene records were listed, according to their gene locus. Users can find the individual gene information, including gene ID, description, gene location, gene orientation, gene length, and gene essentiality (Figure 3A). In addition, users can click the “VC0838” link to redirect to detailed gene information, which includes a list of DEGs in experimental conditions collected in the database (Figure 3C), and the gene co-expression network (Figure 3B). We found that *toxT* is differentially expressed in 28 experiments, which are mainly related to hosting infection and oxidative stress, up-regulated in 9 experiments, and down-regulated in 19 experiments. Additionally, the gene co-expression network suggested that *toxT* is involved in cholera pathogenesis. These observations were consistent with previous findings that ToxT is a transcriptional activator to regulate virulence factors (Baranova et al., 2020; Stone and Withey, 2021).

On the “Operons” page, users can directly search VC0838 to find the corresponding operon, where a table used to provide a summary report, including the Operon ID, and putative operon function. Users can expand the table by clicking the ‘+’ sign before the “Operon ID” to view information on its member genes (Figure 3D).

The “Experiments” page summarized information on 3 Tn-seq projects and 213 transcriptome experiments, including a brief summary of experimental conditions, DEGs (Differentially Expressed Genes), and corresponding reference(s). If users want to know in which experiments VC0838 is differentially expressed, Searching on “Genes” page is a better choice, because “Experiments” page does not provide the summary report in a gene-centric manner.

**Figure 4:**
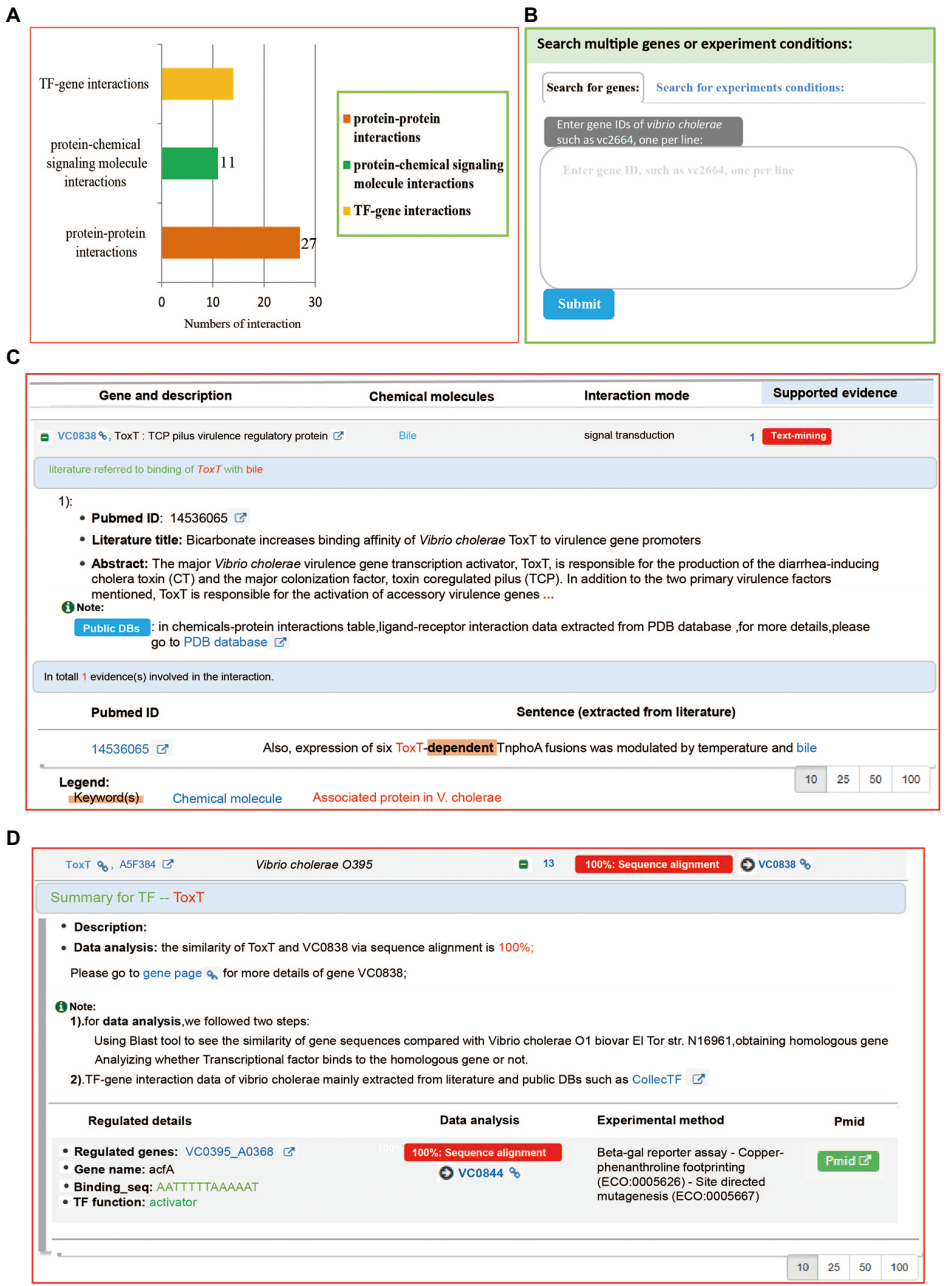
The “Experiments” page. **(A)** A barplot summarizing the interaction data associated with gene VC0803. The Y-axis represents interaction types, and the *X*-axis denotes the number of interactions. **(B)** The search function of the “Interactions” page. Two kinds of search methods: “Search by gene” and “Search by experiment conditions” were provided, allowing users to search what they are interested in. The search results are listed and visualized in an interactive network. **(C)** The protein-chemical signaling molecule interaction. The “VC0838-bile interaction” entry was supported by a sentence extracted from the literature. **(D)** The TF-gene interaction. Users can obtain regulated details they are interested in, including regulated genes, the binding site of transcription factors, and genes.

On the “Interactions” page, all interaction records categorized into four groups according to their interaction type, including protein-chemical signaling molecule interaction, protein–protein interaction, TF (Transcription Factor)-gene interaction, and *Vibrio cholerae*-virus interaction. Users can obtain data of experiment-validated interaction with VC0838. We found that a total 51 interaction records are associated with VC0838, including 27 protein–protein interactions, 11 protein-chemical signaling molecule interactions, and 13 TF-gene interactions (Figure 4A). Regarding details of protein–protein and protein-chemical signaling molecule interactions, Mr.Vc v2 provided reference(s), sentence(s) describing interaction, and keyword(s) extracted from the sentence (Figure 4C). For TF-gene interaction details, a summary was provided that included information on transcription factor function, regulated genes, binding sequence, and experiment method (Figure 4D). Additionally, a search widget was integrated, users can search genes or experimental conditions that they are interested in (Figure 4B), to obtain gene co-expression or experiment-experiment correlation network.

In addition to the above pages, on the “Download” page, all data in Mr.Vc v2 are downloadable as excel files at the “Downloads” page. Users can click the corresponding file name to download the data tables.

## Conclusion

In this study, we introduced Mr.Vc v2, an updated version of the online database of curated omics data and annotation information. Updates since the last version include increased numbers of RNA-seq experiments/samples, experimental validated interactions, and results of data analysis. So far, Mr.Vc v2 includes 3,998 genes, 2,366 operons, 213 transcriptome experiments, 3 Tn-seq projects, and 1,316 entries of experimentally validated interaction. Additionally, through data analysis, Mr.Vc v2 obtained 56,739 DEGs, 270,129 entries of gene co-expression, and 11,990 entries of experiment-experiment correlation. We believe that Mr.Vc v2 is expected to be a highly useful and important database for biologists and bioinformaticians studying *Vibrio cholerae*. In the future, we aim to update Mr.Vc v2 regularly to provide up-to-date content and include more functionalities.

## Data availability statement

Publicly available datasets were analyzed in this study. This data can be found here: http://mrvcv2.biownmc.info/download/.

## Author contributions

ZZ, MY, and HZ designed the study. ZZ and GC collected the data. ZZ analyzed the data prepared and the first draft. WH reviewed and edited the final draft. All authors approved the final submission.

## Funding

This work was supported by the National Natural Science Foundation of China (61672386), the Academic Support Project for Top-notch Talents in Disciplines (Majors) of Universities in Anhui Province (gxbjZD2022042), and the Key Research and Development Plan of Anhui Province, China (2022a05020011).

## Conflict of interest

The authors declare that the research was conducted in the absence of any commercial or financial relationships that could be construed as a potential conflict of interest.

## Publisher’s note

All claims expressed in this article are solely those of the authors and do not necessarily represent those of their affiliated organizations, or those of the publisher, the editors and the reviewers. Any product that may be evaluated in this article, or claim that may be made by its manufacturer, is not guaranteed or endorsed by the publisher.

## Footnotes

^1^ https://www.ebi.ac.uk/ena

^2^ https://www.ncbi.nlm.nih.gov/sra

^3^ https://www.ncbi.nlm.nih.gov/bioproject

^4^ https://pubmed.ncbi.nlm.nih.gov/

^5^ https://www.ebi.ac.uk/ena

^6^ https://www.ncbi.nlm.nih.gov/sra

^7^ https://github.com/enasequence/enaBrowserTools

^8^ https://ncbi.github.io/sra-tools/

^9^ http://www.bioinformatics.babraham.ac.uk/projects/fastqc/

^10^ http://mrvcv2.biownmc.info/operons/OP437

^11^ http://mrvcv2.biownmc.info/help/

^12^ https://www.ncbi.nlm.nih.gov/gene/

^13^ http://mrvcv2.biownmc.info/genes/
